# Direct Prediction of EPR Spectra from Lipid Bilayers: Understanding Structure and Dynamics in Biological Membranes

**DOI:** 10.1002/cphc.201800386

**Published:** 2018-06-19

**Authors:** Andrea Catte, Gaye F. White, Mark R. Wilson, Vasily S. Oganesyan

**Affiliations:** ^1^ School of Chemistry University of East Anglia Norwich NR4 7TJ UK; ^2^ Department of Chemistry Durham University, Lower Mountjoy South Road Durham DH1 3 LE UK

**Keywords:** biological membranes, EPR spectroscopy, molecular dynamics simulations, spin probes, cholesterol

## Abstract

Of the many biophysical techniques now being brought to bear on studies of membranes, electron paramagnetic resonance (EPR) of nitroxide spin probes was the first to provide information about both mobility and ordering in lipid membranes. Here, we report the first prediction of variable temperature EPR spectra of model lipid bilayers in the presence and absence of cholesterol from the results of large scale fully atomistic molecular dynamics (MD) simulations. Three types of structurally different spin probes were employed in order to study different parts of the bilayer. Our results demonstrate very good agreement with experiment and thus confirm the accuracy of the latest lipid force fields. The atomic resolution of the simulations allows the interpretation of the molecular motions and interactions in terms of their impact on the sensitive EPR line shapes. Direct versus indirect effects of cholesterol on the dynamics of spin probes are analysed. Given the complexity of structural organisation in lipid bilayers, the advantage of using a combined MD‐EPR simulation approach is two‐fold. Firstly, prediction of EPR line shapes directly from MD trajectories of actual phospholipid structures allows unambiguous interpretation of EPR spectra of biological membranes in terms of complex motions. Secondly, such an approach provides an ultimate test bed for the up‐to‐date MD simulation models employed in the studies of biological membranes, an area that currently attracts great attention.

## 1 Introduction

Phospholipid bilayers have been extensively investigated and employed as models of biological membranes over the past decades.^1^ Knowledge of how molecular interactions control molecular order and dynamics are crucial for understanding the role that different lipids play in vital life processes in biological membranes. It is known that the dynamical structure of lipid membranes is very complex. This area of research attracts considerable interest and a vast variety of spectroscopic techniques have been employed to various model bilayer systems, for example nuclear magnetic resonance (NMR),^2^ time‐resolved fluorescence,^3^ fluorescence correlation spectroscopy^4^ and fluorescence resonance energy transfer (FRET).^5^ Such studies are aimed at elucidation the physical mechanisms that are responsible for structural organisation, dynamics and their relation to biological functions of phospholipids in cell membranes.

Out of all the biophysical techniques now being brought to bear on studies of membranes continuous wave (CW) electron paramagnetic resonance (EPR) of nitroxide spin probes was the first to provide information about mobility and ordering in lipid membranes and lipid bilayer systems.^6^ EPR is a ‘fast’ spectroscopic technique that can resolve molecular re‐orientational dynamics of the introduced spin probe on sub‐nanosecond timescales and has been widely used to study membrane structure and dynamics.^7^

Structurally variable nitroxide spin labels can probe different parts of the bilayer and also be attached to embedded peptides and proteins. Most commonly, EPR spin probes containing nitroxide groups at different positions in the fatty acid chain, such as 1‐palmitoyl‐2‐stearoyl‐(n‐doxyl)‐*sn*‐glycero‐3‐phosphocholines (n‐PC spin probes), and in the cholesterol (CHOL) head group, such as 3β‐doxyl‐5α‐cholestane (CSL), have been employed to study the structure of lipids7a,7b,7d,7e and their interaction with membrane proteins.7c In early studies, Hubbell and McConnell introduced the application of spin probes with EPR spectroscopy to investigate the dynamics of various parts of fatty acid chains in phospholipid bilayers.7d Freed and co‐workers used a combination of multi‐frequency CW EPR and 2D‐ELDOR spectroscopy with theoretical modelling of EPR spectra in extensive studies of different lipid phases formed in model lipid membranes.^8^ They also studied plasma membrane vesicles of RBL‐2H3 mast cells,7e,^9^ lipid‐protein interactions^10^ and coexisting lipid domains on the ternary phase diagrams including the determination of the tie‐lines.^11^ Smirnov and co‐workers have employed pH‐sensitive nitroxide based spin probes for studying local electrostatic properties of lipid bilayers and proteins.^12^ Marsh and co‐workers have applied EPR spectroscopy with a variety of nitroxide spin probes to study the polarity and permeation profiles of water, oxygen and ions into DPPC lipid membranes without and with cholesterol.^13^

By careful line shape analysis, with the aid of rigid rod models and fitting of spectra, detailed information about ordering and motions of the lipids in the membrane can be elucidated.11a Such an approach, however, relies on the use of simple models (e. g. particle in an anisotropic potential) for molecular movements and does not reflect the full complexity of actual molecular structure and its impact on motions. Moreover, with the application of multiple adjustable parameters the fitting and therefore interpretation of EPR line shapes might not be unique in this approach.^14^

Here, we report the first time prediction of EPR spectra from lipid bilayers doped with paramagnetic spin probes directly and entirely from the results of fully atomistic MD simulations using a trajectory‐based MD‐EPR simulation methodology.^14^, ^15^ Our approach takes advantage of the recent dramatic improvements in the modelling of complex molecular and bio‐molecular systems at the atomistic level,15a,^16^,^17^ The advantage of our approach is two‐fold. Firstly, it provides detailed analysis of molecular motions and organisation in lipid bilayers (at picosecond snapshot resolution), demonstrating the extreme sensitivity of the EPR signal to changes in local membrane structure and dynamics. Secondly, a combined MD‐EPR methodology serves as an ideal test bed for advanced computational models for lipid bilayer simulations thus facilitating their improvement. We use our approach to demonstrate how spin probes report on the structure and dynamics of the surrounding lipids by explicitly accounting the motional complexity of both.

In this work we combine all‐atom (AA) MD simulations with variable temperature CW EPR of three structurally different nitroxide paramagnetic spin probes, namely, 16‐PC, 5‐PC and CSL doped in 1,2‐dipalmitoyl‐*sn*‐glycerophosphatidylcholine (DPPC) lipid bilayers in order to predict EPR spectra, that are highly variable in line shapes depending on the bilayer composition, type of spin probe, and temperature value. We also investigate the phase behaviour of DPPC in the absence and in the presence of cholesterol (CHOL).

## **Experimental Section**

### MD Simulations

The study has been performed within the framework of the latest version of the Stockholm lipids (Slipids) force field^18^ by comparing the predicted EPR spectra with the experimental ones. A total of thirty large scale AA MD simulations have been performed and used for direct simulation of EPR spectra over a range of temperatures between 298 K and 333 K. The simulation models use pure DPPC membranes contained 488 lipids and DPPC/CHOL mixtures with 408 and 180 molecules of DPPC and CHOL, respectively. Each lipid membrane contains 2 % molar concentration of spin probes, corresponding to 12 spin probe molecules in a bilayer (6 spin probe molecules per leaflet).

Details of the preparation of the lipid bilayer systems contacting different spin probes of appropriate concentration and in the absence and presence of 30 % molar concentration of CHOL are provided in Supporting Information. Each DPPC lipid bilayer was solvated with 30 water molecules per lipid and ionized with a proper amount of Na^+^ and Cl^‐^ ions to reach a physiological ionic strength of 150 mM. Total number of atoms, including water and ions, reached values of approximately 132,000 atoms for systems without and with CHOL. All‐atom (AA) MD simulations were performed using Gromacs version 4.5.5.^19^ A refined version of the CHARMM36 force field in Gromacs, which is known as the Slipids force field, was used to describe the lipids.^18^ Non‐bonded van der Waals and electrostatics interactions were truncated using a cut‐off distance of 12 Å. The PME treatment of long range electrostatic interactions was employed. Temperature and pressure for all simulated DPPC and DPPC:CHOL lipid bilayers were stabilized at different temperatures (ranging from 283 K to 333 K) and 1 atm using a Nosé‐Hoover thermostat^20^ and a Parrinello‐Rahman barostat,^21^ respectively. Coordinate trajectories were updated every 20 ps.

### Parametrisation of Nitroxide Spin Probes

The General Atomic force field (GAFF) parameters for a CSL spin probe have been previously developed by us[17c] and are compatible with the Slipids force field. Quantum chemical calculations of 5‐PC and 16‐PC spin probes were performed with the Gaussian09^22^ software package, to obtain partial charges using the restricted electrostatic potential approach (RESP) carried out with the RED software.^23^ Force‐field parameters for the new atom types of the nitroxide moiety in 5‐PC and 16‐PC (the unsaturated carbon atoms of the nitroxide ring, the saturated carbon atoms of the nitroxide ring, the nitrogen and the oxygen) were taken from a combination of geometry optimization calculations in the gas phase and previous calculations. Equilibrium bond lengths and angles were taken directly from minimized energy structures. Force constants were interpolated using the reference values in the AMBER99 force field^24^ and the quantum mechanical calculations of Barone and co‐workers.^25^ New torsional parameters were calculated as described previously.17c

### Autocorrelation Functions and Calculation of Effective Rotational Correlation Times

The autocorrelation function of each molecular vector $\vec{v}\left(t\right)$ discussed in the text is calculated from an MD trajectory according to the following expression [Eq. (1)]:

(1) $$C\left(t\right)=\left\langle \int_{0}^{\infty }P_{2}\left(\vec{v}\left(\tau \right)\cdot \vec{v}\left(t+\tau \right)\right)d\tau \right\rangle$$

where $P_{2}\left(t\right)$ is the second order Legendre polynomial and the bracket denotes the average taken over the orientation distribution, time and the number of available molecules. A ‘sliding time window’ approach was used for time averaging.15a Effective rotational correlation times of the vectors were calculated from a bi‐exponential fitting of the respective autocorrelation functions using Equation (2):

(2) $$C\left(t\right)=\left(1-S_{0}^{2}\right)\left(w_{1}\exp\left(-t/\tau_{1}\right)+w_{2}\exp\left(-t/\tau_{2}\right)\right)+S_{0}^{2}$$

in which $S_{0}^{2}$ is the square of the generalized order parameter $S_{0}$ of the vector. In the case of *z* or *y* magnetic axes of the 5/16‐PC and CLS spin probes, respectively, it is defined as follows [Eq. (3)]:11a

(3) $$S_{0}=\left\langle \frac{1}{2}\left(3\cos^{2}\theta -1\right)\right\rangle$$

where *θ* is the angle between the bilayer normal of the top or bottom leaflet (director) and the axis. The effective correlation time is related to the individual correlation times and the weights$\;w_{i}$ according to the following relationship: $\tau_{eff}=w_{1}\tau_{1}+w_{2}\tau_{2}$ .

### MD‐EPR Simulation Approach

A trajectory based method that employs the numerical solution of the Stochastic Liouville Equation (SLE) in the Langevin form for the spin density matrix has been used for the simulation of CW EPR line shapes.^14^, ^15^,^26^ This method has been successfully applied previously to liquid crystals and proteins.15a,^17^ A program, developed and described previously by one of us,15a,26b was used for simulations and subsequent analysis of EPR spectra. In the program single concatenated MD trajectories are used to calculate the variation in time of the averaged transverse magnetisation and, eventually, the EPR line shapes.^14^, ^15^

**Figure 1:**
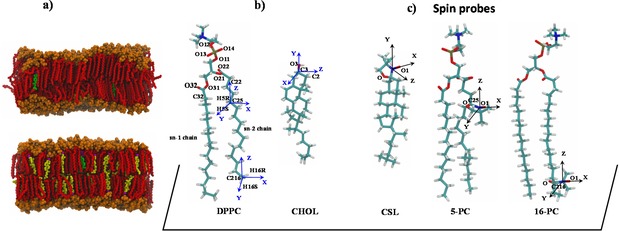
a) Side views of snapshots from 100 ns AA MD simulations of DPPC (top) and DPPC:CHOL (bottom) lipid bilayers doped with CSL spin probe molecules at 298 K. DPPC polar head groups and hydrophobic tail groups are shown in orange and red, respectively. CHOL hydroxyl group and sterol ring/alkyl chain are shown in magenta and yellow, respectively. CSL spin probe nitroxide moiety and sterol ring/alkyl chain are shown in purple and green, respectively. Water, hydrogens and ions are not shown for clarity. b) Molecular structures of DPPC and CHOL; c) Molecular structures of three employed spin probes. For the spin probes the magnetic axes of the nitroxide moieties are indicated in black. For DPPC and CHOL associated molecular frames are given in blue.

Relatively long total MD trajectories generated in this work allowed the simulation of EPR spectra directly by propagation of the spin density matrix along the entire sampling times without further approximations. At each time increment the propagation of the density matrix was achieved analytically in Hilbert space using the eigenvalues and eigenvectors of the Spin‐Hamiltonian.15b Statistical averaging was achieved by the “sliding time window technique” allowing the use of single MD trajectories.15a Further details are provided in the Supporting Information. The EPR spectral line shapes of nitroxide spin labels are determined entirely by the variation with time of two angles that define the orientation of the applied magnetic field to the principle axis of the nitroxide group. Therefore, the orientational history of the magnetic axes in the fixed frame of the simulation box is calculated and processed. For each spin probe the z axis of the nitroxide ring (coincident with the direction of p_z_‐orbital of N) is calculated from the cross‐product of the unit vectors of two N−C bonds of the ring (see Figure 1). The x axis is calculated as a projection vector of the N−O bond on the nitroxide plane (defined by the C−N−C atoms) and the y axis is taken as a cross‐product of the z and x vectors.

### Materials and Sample Preparation

Dipalmitoylphosphatidylcholine (DPPC) and the spin‐labelled lipids 1‐palmitoyl‐2‐(16‐ doxyl stearoyl) phosphatidylcholine (16‐PC) and 1‐palmitoyl‐2‐(5‐doxyl stearoyl) phosphatidylcholine (5‐PC) were purchased from Avanti Polar Lipids (Alabaster, AL). Cholesterol and the 3β‐doxyl‐5α‐ cholestane (CSL) were from Sigma (UK). Measured stock solutions of the lipids and spin‐labels in chloroform/methanol (2 : 1 v/v) were mixed in a glass bottle, evaporated to dryness under a stream of N_2_ and further dried in a vacuum desiccator for 3 hrs.7b The concentration of spin‐label was circa 2.0 mol % of the lipids. Phosphate buffered saline, warmed to 60 °C, was added to the lipid film and the mixture vortexed vigorously to give a 40 mM lipid suspension. Samples were incubated in an anaerobic glovebox for 1 hour and transferred to 1.1 mm (inner diameter) capillaries under anaerobic conditions.11b Each capillary was sealed with capillary wax (Hampton Research).

### Variable Temperature EPR Measurements

EPR spectra were measured using an X‐band Bruker EMX spectrometer equipped with the digital temperature control system (ER4131VT) for high temperature measurements using a heated flow of nitrogen gas. The following conditions were used: microwave frequency of 9.55 GHz; microwave power of 2 mW; modulation frequency of 100 kHz; modulation amplitude of 1.0 G. For each temperature samples were equilibrated for 5 minutes before taking the measurement. Variable temperature measurements were performed with the tolerance <0.1 K.

## 2 Results and Discussion

Examples of snapshots of MD simulations of DPPC and DPPC:CHOL lipid bilayers together with the structures of DPPC, CHOL and three spin probes employed in this study are presented in Figure 1. Pure DPPC lipids exhibit a gel or solid ordered (S_o_) phase to liquid crystalline (L_α_) phase transition (i. e. liquid disordered, L_d_, phase) at 41 °C, which is also known as the chain melting temperature. The presence of CHOL induces an ordering of the acyl chains of DPPC and other saturated lipids leading to the formation of a liquid ordered (L_o_) phase.

This structural change is attributed to the well‐known condensing effect of CHOL.^5^ CHOL broadens the gel‐liquid phase transition of DPPC by preventing packing at low temperatures, disrupting gel formation, and inducing order in the liquid phase at higher temperatures.^27^ The coexistence of L_d_ and L_o_ phases has also been observed in giant unilamellar vesicles containing CHOL and two types of lipids. An example of the equilibrated structures of DPPC and DPPC : CHOL lipid bilayers using Slipids force fields doped with CSL spin probes at 298 K is presented in Figure 1a. The difference in the bilayer thickness between the two systems is due to the ordering effect of CHOL,^28^ which, as a consequence of the high CHOL concentration (30 mol %), induces the formation of the L_o_ phase over the temperature range that was simulated (298 K–333 K).

For both pure DPPC and DPPC : CHOL lipid bilayers, the calculated structural properties (e. g. simulation snapshots and the deuterium order parameters profiles for DPPC) are presented in the Supporting Information (Figures S1–S3). They indicate an increase of the general disorder in DPPC lipid bilayers with increasing temperature. They also confirm that the DPPC lipid bilayer in the absence of CHOL is in the S_o_ phase at 298 K and below.

Upon approaching the phase transition temperature of DPPC (*T*_m_ : 314 K) the lipid bilayer becomes more fluid with the observation of the pure L_d_ phase at 318 K. In the presence of CHOL the L_o_ phase coexisting with either a S_o_ phase or a L_d_ phase is observed at temperatures below or above *T*_m_, respectively. These simulation results are in good agreement with the phase diagram determined using 2D‐ELDOR.8a

### 2.1 Comparison Between Predicted and Experimental EPR Spectra in the Presence and Absence of Cholesterol

X‐band CW EPR is highly sensitive to molecular motions in the time range of 10^−7^–10^−11^ s,6a making it particularly suitable as a technique to study the dynamics and order in lipid bilayers by employing structurally different spin probes accessing different parts of the membrane. The magnetic tensors **g** and **A**, with **g** defining the interaction of the electron spin of the probe with the external magnetic field and **A** the hyperfine coupling with the nuclear spin of ^14^N, are both anisotropic leading to a strong dependence of the EPR resonances on the direction of the principle magnetic axes relative to the external magnetic field.

As a result, the EPR line shapes are sensitive to both the dynamics and the order of the probe. These shapes can range from completely averaged three narrow lines in the case of fast isotropic motion to broad asymmetric lines in the case of strongly immobilised spins. A variety of different line shapes can be observed in between these two extremes depending on the effective correlation time of the re‐orientational dynamics of the probe and the degree of motional constrains imposed on it by the immediate environment. At X‐band (9.5 GHz) the dominant contribution to the anisotropy comes from the hyperfine interaction resulting in the EPR spectra consisting of three hyperfine coupling lines (see Equation. S2 in the Supporting Information).

In this study the concentration of doped spin probes was kept at ∼2 %. Previously it has been shown that such a low concentration does not affect the phase behaviour in DPPC and other lipid bilayer systems.7b A relatively large size of the lipid bilayer has permitted us to employ 12 probes (6 at each leaflet) in MD simulation runs of 100 ns duration. In each MD run the resulting dynamical trajectories of individual probes were then concatenated into a relatively long 1.2 μs trajectory.

EPR line shapes include contributions from the overall and internal motions of the nitroxide spin probes in the oriented environment of the phospholipids. The fast motion is mainly controlled by the inter acyl chain dynamics, whereas the slower motion contribution is associated with the overall tumbling of the lipid molecules. The addition of CHOL impacts on both types of motion, although the effects on each can be different depending on how CHOL interacts with both DPPC and spin probes.

### 2.2 Lipid Bilayers Doped with 5‐PC Spin Probes

**Figure 2:**
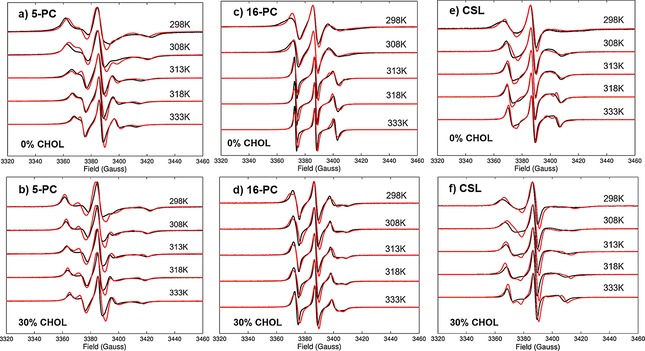
Comparison between predicted from MD (red lines) and experimental (black lines) EPR spectra of DPPC and DPPC:CHOL lipid bilayers doped with 5‐PC [(a) and b)], 16‐PC [(c) and (d)] and CSL [(e) and (f)] spin probes at different temperatures. All spectra are normalised to their maximal intensity. Additionally, area‐normalised spectra (normalised by the value calculated by double integration of the EPR line shape) are compared in Figure S4 in the Supporting Information.

Comparison between predicted from MD and experimental EPR spectra for pure DPPC and DPPC with 30 % CHOL both doped with 5PC are shown in Figure 2a and 2b, respectively. EPR spectra predicted from MD trajectories are in very good agreement with the experimental ones reproducing, at each temperature, all the characteristic features of the line shapes.

EPR spectra corresponding to 5‐PC are characterised by the broadest line shapes among the three spin probes and are highly sensitive to the variations in temperature. Such characteristic line shapes are associated with the restrained motion of the spin probe in the partially oriented dynamical environment of the lipids. As one can see from Figure 1c, the nitroxide rings of both the 5‐PC and 16‐PC probes lie approximately perpendicular to the acyl chain. The magnetic *z*‐axis is oriented perpendicular to it and has orientation along the membrane normal averaged over the trajectory snapshots. The outer peak positions in the EPR hyperfine coupling lines are sensitive to both the order and the dynamics of the magnetic *z*‐axis. The order parameters of both 5‐PC and 16‐PC can be estimated directly from the difference between the outer peaks of the line shape following Hubbell and McConnell [Eq. (4)]:7d

(4) $$S_{0}=\frac{\left(A{{}^{'}}_{\left|\right|}-A{{}^{'}}_{⊥}\right)}{\left(A_{zz}-1/2\left(A_{xx}+A_{yy}\right)\right)}$$

where $A_{ii}$ are the principle components of the hyperfine coupling tensor of the spin probe and $A{{}^{'}}_{\left|\right|}$ and $A{{}^{'}}_{⊥}$ are effective inner and outer hyperfine splittings, respectively, seen in the motional EPR spectrum.

The effective hyperfine splitting parameter serves as a measure of nitroxide mobility with larger values corresponding to lower mobility.^29^ As one can observe in Figure 1a and 1b the positions of outer peaks are well reproduced in MD predicted EPR spectra across the simulated temperature range.

The values of the order parameter estimated from both experimental ($S_{0}^{b})\;$ and predicted ($S_{0}^{c})$ EPR spectra using Equation (4) are given in Table 1. They are compared to the corresponding values $\left(S_{0}^{a}\right)\;$ obtained from MD. In all three cases the corresponding values in the presence of CHOL are given in brackets. It is interesting to note that an excellent agreement is observed between the order parameter values estimated from MD and those calculated from the spectra using Equation (4) for temperatures 313 K–333 K while some disagreements are seen for lower temperatures. The latter reflects the fact that at temperatures 298 K and 308 K the internal dynamics of the probes are deep within the slow motion regime, which is confirmed by the correlation times obtained from MD data. This results in the overestimation of the order parameters by Equation (4), which has been derived under the assumption of the partial averaging of the magnetic tensor components due to fast local motions.

**Table 1:** Effective correlation times and order parameters of the magnetic axes of 5‐PC, 16‐PC and CSL spin probes.

*T* [K]	$\tau_{eff}^{y}$ [ns]	$\tau_{eff}^{z}$ [ns]	$S_{0}^{a}$	$S_{0}^{b}$	$S_{0}^{c}$	$\tau_{eff}^{y}$ [ns]	$\tau_{eff}^{z}$ [ns]	$S_{0}^{a}$	$S_{0}^{b}$	$S_{0}^{c}$	$\tau_{eff}^{y}$ [ns]	$\tau_{eff}^{z}$ [ns]	$S_{0}^{a}$
	5‐PC					16‐PC					CSL		
298	12.5 (12.5)^[a]^	16.4 (19.8)	0.65 (0.73)	0.83 (0.83)	0.72 (0.79)	3.9 (4.2)	4.4 (3.3)	0.20 (0.30)	0.35 (0.31)	0.25 (0.36)	13.3 (14.0)	11.6 (5.8)	0.55 (0.78)
308	7.7 (10.5)	5.9 (13.9)	0.48 (0.71)	0.76 (0.79)	0.67 (0.72)	1.1 (1.0)	2.0 (1.8)	0.17 (0.24)	0.27 (0.27)	0.27 (0.31)	7.9 (7.6)	4.4 (3.5)	0.39 (0.73)
313	4.7 (10.8)	5.3 (7.6)	0.49 (0.65)	0.57 (0.74)	0.64 (0.70)	0.5 (0.8)	0.7 (0.7)	0.14 (0.20)	0.12 (0.27)	0.18 (0.27)	4.8 (6.9)	2.3 (2.1)	0.39 (0.66)
318	3.2 (9.6)	3.1 (6.5)	0.51 (0.66)	0.49 (0.70)	0.52 (0.68)	0.4 (0.8)	0.5 (0.7)	0.10 (0.17)	0.10 (0.27)	0.11 (0.28)	4.5 (4.2)	2.2 (1.9)	0.36 (0.65)
333	2.6 (2.4)	2.6 (6.1)	0.41 (0.52)	0.41 (0.57)	0.43 (0.59)	0.2 (0.2)	0.3 (0.3)	0.09 (0.17)	0.07 (0.18)	0.11 (0.18)	2.2 (2.6)	1.0 (0.7)	0.34 (0.61)

$S_{0}^{a}$ represents order parameters for the *z*‐axes of 5‐PC and 16‐PC and *y*‐axis of CLS spin probes (see Figure 1) determined from the fitting of associated autocorrelation functions using Equation (2). Results of the fitting are provided in Tables S1 and S2 of the Supporting Information; $S_{0}^{b}$ and $S_{0}^{c}$ represent order parameters of the *z*‐axes of 5‐PC and 16‐PC estimated from experimental and predicted from MD EPR spectra, respectively, using Equation (4); [a] values in parentheses are estimated for DPPC:CHOL mixtures.

**Figure 3:**
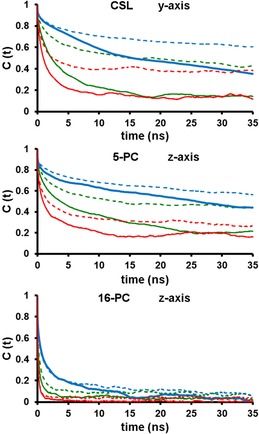
Rotational autocorrelation functions of the *y* and *z* magnetic axes of CSL and 5/16‐PC spin probes, respectively, calculated at 298 K (skyblue), 313 K (green) and 333 K (red). In each subpanel autocorrelation functions from spin probes in DPPC and the DPPC:CHOL lipid bilayer mixture are presented as solid and dashed lines, respectively.

The autocorrelation functions for the magnetic *z*‐axis of 5‐PC obtained from MD simulations using Equation (1) are shown in Figure 3 for selected temperatures. They can be approximated by a sum of exponential decays, with correlation times that can be attributed to different rotational motions. We observe a fast decay, in the regime of a few hundreds of picoseconds, followed by a slower decay mode of hundreds of picoseconds to several nanoseconds depending on the probe and temperature. Different contributions were estimated by performing a bi‐exponential fit of the total auto‐correlation curve using Equation (2) with the adjusted parameters summarised in Tables S1 and S2 in the Supporting Information. The fast decay is attributed to the local rotational motions of each probe (e. g. internal dynamics of the acyl chain). The second decay time is attributed to the slow reorientation motion of the restricted local environment of the spin probe imposed by the surrounding phospholipids. The values of effective rotational correlation time and order parameter for the *z*‐axis of 5‐PC calculated at different temperatures are given in Table 1 with the corresponding values for the DPPC:CHOL mixture shown in brackets.

According to Table 1 in the case of pure DPPC the effective correlation time for *z* ‐magnetic axis of 5‐PC ranges between 16.4 ns and 2.6 ns for 298 K and 333 K temperatures, respectively, and correspond to the so‐called slow motional regime. As expected, upon increasing the temperature both the correlation time and the order parameter of 5‐PC are progressively reduced, in line with the decreased distance between the outer peak positions of the EPR line shapes (Figure 2a). On addition of CHOL experimental spectra undergo noticeable changes by becoming broader at each temperature (Figure 2b). This effect is well reproduced in the predicted EPR spectra. The average order parameter of 5‐PC is increased by approximately a factor of ∼1.3 for all temperatures. The effective correlation times are also increased in the presence of CHOL from 16.4 ns to 19.8 ns and from 2.6 ns to 6.1 ns for 298 K and 333 K, respectively. Increases in both correlation time and order parameter contribute to the broadening of the EPR line shapes. Similar to the case of pure DPPC doped with 5‐PC, the temperature variation of predicted outer peak positions in EPR spectra obtained from the MD trajectories for the DPPC:CHOL mixture is in very good agreement with experimental measurements confirming the accuracy of MD simulation model employed in this study.

### 2.3 Lipid Bilayers Doped with 16‐PC Spin Probes

Comparisons between predicted from MD and experimental EPR spectra in the case of doped 16‐PC spin probe are presented in Figure 2c and 2d. As with 5‐PC, the nitroxide ring of 16‐PC has a perpendicular orientation to the acyl chain.

However, its position, significantly closer to the end of the chain, provides a higher degree of motional and orientational flexibility. As a result, the EPR line shapes of 16‐PC become much narrower compared to 5‐PC. The spectra predicted directly from MD for pure DPPC doped with 16‐PC show excellent agreement with experiment across the 298 K–333 K temperature range (Figure 2c). Similar to the case of 5‐PC, Table 1 provides a comparison among the values of the order parameter of the *z*‐axis of 16‐PC estimated from both experimental and predicted EPR spectra and calculated from MD trajectory. At 298 K the dynamics of 16‐PC is characterised by a relatively broad EPR line shape with the correlation time of 4.4 ns and the order parameter $S_{0}^{a}$ =0.20. A sudden characteristic change in the EPR line shape is predicted at 313 K, corresponding to the S_o_‐L_d_ phase transition, in full agreement with the EPR experimental observation (Figure 2c). This transition is associated with the increased motions of the acyl chains of DPPC leading to the narrowing of three hyperfine coupling lines in the EPR spectrum. Indeed, according to Table 1 the correlation time for magnetic *z*‐axis drops from 2.0 ns at 308 K to 0.7 ns at 313 K. At the same time the value of the corresponding order parameter is decreased from 0.17 to 0.14. The values of *τ* and *S_0_* calculated from MD are in good agreement with the previously determined values of 16‐PC in pure DPPC reported by Freed and co‐workers and obtained from the fitting of variable temperature (VT) EPR spectra using a combination of microscopic order macroscopic disorder (MOMD) and slowly relaxing local structure (SRLS) simulation models in conjunction with the rotational dynamics of a rigid rod in an axial ordering potential.^30^

For instance, the effective correlation times across the phase transition region estimated by Freed et al.^30^ and calculated in this study are 2.7 ns and 2.0 ns for 308 K, respectively, and 0.6 ns and 0.5 ns for 318 K, respectively, while the corresponding order parameters compare at 308 K as 0.23 and 0.17, respectively, and at 318 K as 0.08 and 0.10, respectively. However, in our approach all contributions from molecular motions of 16‐PC into the EPR line shapes are explicitly accounted for by the atomistic MD trajectory. Upon further increases in temperature the dynamics of 16‐PC in DPPC becomes faster and less constrained reaching an EPR line shape that is characteristic of fast motion in a weakly restraining media ($S_{0}^{a}\;$ =0.09).

The situation is different for the MD‐EPR simulation results of the DPPC mixture with CHOL (Figure 2d). The predicted line shapes are consistently broader across the temperature range with no definite changes seen at 313 K. A small splitting of the low field feature is visible in the predicted line shapes at 298 K and 308 K indicating slight overestimation of the order of the *z*‐axis of the nitroxide moiety in 16‐PC by the employed MD simulation model. Interestingly, although the calculated correlation times of the *z*‐axis are progressively reduced from 298 K to 333 K they appear marginally similar to those calculated in the case of pure DPPC system. In contrast, with the addition of CHOL the order parameter of the *z*‐axis becomes significantly higher (by a factor of 1.5 for 298 K–313 K and a factor of 2 for 318 K and 333 K). The increased value of *S_0_* is responsible for the observed broadening of both *I*=1 and *I*=−1 hyperfine coupling lines resulting in the noticeable split seen in the high field arm of the spectrum (at ∼3404 Gauss).

### 2.4 Lipid Bilayers Doped with CSL Spin Probes

A comparison between predicted from MD and experimental EPR spectra for CSL spin probe in DPPC and DPPC:CHOL mixture is presented in Figure 2e and 2 f. In both cases simulated and experimental spectra are again in good agreement with each other. The EPR line shapes of CSL are significantly different from those of 5‐PC and 16‐PC due to the structural differences between the cholestane and phospholipid based probes. In particular, the plane of the nitroxide head group in CSL lies along the main axis of the probe, which puts its orientation perpendicular to the bilayer with the orientation of the magnetic *y*‐axis being the closest to the membrane normal. Effective correlation times and order parameters for both *y* and *z* axes are given in Table 1. The axial rotation of the probe along its main axis averages the resonances in the *xz* magnetic plane.

For both pure DPPC and DPPC:CHOL mixture at 298 K the motions of both axes are slow (11.7 ns and 11.6 ns for *x* and *z* axes, respectively) and resonances along all orientations are well resolved in the EPR spectrum resulting in a characteristic almost zero plateau observed along the high field hyperfine coupling line in the EPR spectrum.

Upon temperature increase the correlation times for all three magnetic axes are reduced with the strongest effect experienced by the *z* and *x* axes. This leads to the partial averaging of the resonances in the *xz*‐plane (observed at temperatures 308 K and 313 K) and then complete averaging at 333 K with the sharp derivative type features emerging in the EPR line shape. Additionally, the tilting dynamics of CSL leads to partial averaging between the resonances along the *y* and *z* axes. The degree of this averaging is increased with temperature and is directly related to the value of the order parameter of the *y*‐axis (tilting motions of the label relative to its preferred orientation caused by surrounding phospholipids). The S_o_‐L_d_ phase transition is still visible in both experimental and simulated VT EPR spectra of CSL in pure DPPC, although this is less pronounced compared to the spectra for 16‐PC. Similarly to 16‐PC, in the DPPC:CHOL mixture doped with CSL the effective correlation times remain very close to the ones calculated for pure DPPC at all temperatures, while the corresponding values of the order parameter are significantly increased (see Table 1).

As one would expect, the changes in EPR line shapes of CSL become more gradual in the presence of CHOL. The outer positions of low and high field features in the DPPC:CHOL mixture are shifted to the left and right, respectively, compared to the ones in the spectra of pure DPPC. This is the result of an almost two‐fold increase in the order parameter of the CSL probe at all five temperatures (see Table 1) upon addition of 30 % of CHOL.

It is important to note that the predicted from MD VT EPR spectra using Slipids force fields correctly reproduce all the essential features in the EPR line shapes that are characteristic to the motions of 5‐PC, 16‐PC and CSL spin probes, both in pure DPPC bilayer and in the DPPC/CHOL mixture. In particular, the model correctly predicts: i) the positions of the outer and inner peaks in the spectra and their changes with the temperature (most prominently observed in the case of 5‐PC); ii) observation of a characteristic change in the EPR line shape during the phase transition at ∼313 K in pure DPPC which is obscured by the presence of CHOL, in full agreement with experiment (most prominently seen for 16‐PC) and iii) prediction of a characteristic broad feature in the high field region with almost zero plateau observed at low temperatures for CLS spin probe. At the same time some discrepancies are present between the predicted and experimental spectra, although in several instances the agreement between the prediction and experiment appears to be better than between the fitted and experimental spectra typically reported in the literature. Apart from the accuracy of the employed MD simulation model itself, the discrepancies are attributed to the limited lengths of the dynamical trajectories employed.^31^ In order to illustrate this point, we have performed a simulation of EPR spectrum of 5‐PC probe in DPPC at 308 K using a different 1.2 μs MD trajectory. The EPR spectra predicted from two trajectories of the same length are compared in Figure S5 showing small but still noticeable discrepancies between the two. In addition, Figure S6 demonstrates the convergence of the simulated EPR line shape based on the use of different lengths of the MD trajectory, namely 400 ns, 600 ns, 800 ns, 1000 ns and 1200 ns, corresponding to the use of 4, 6, 8, 10 and 12 probes, respectively.

### 2.5 Direct Versus Indirect Impact of Cholesterol on the Order and Dynamics of Spin Probes in DPPC

**Figure 4:**
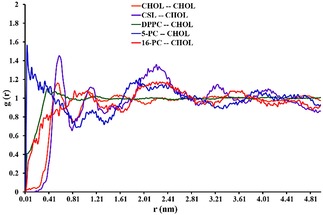
RDFs of the distance between the P and O atoms of 5/16‐PC and CSL, respectively, and O atom of CHOL, projected onto the bilayer plane.

Here, we analyse the effect of CHOL on the molecular motions of three structurally different spin probes and link them with the changes in the relevant EPR line shapes. Interactions with CHOL molecules are analysed by the comparison among the Radial Distribution Functions (RDFs) of the distances between the P and O atoms of 5/16‐PC and CSL, respectively, and the O atom of CHOL, projected onto the bilayer plane, shown in Figure 4. As one can see in the cases of 5‐ and 16‐PC RDFs have significant intensity between 0 and 0.4 nm, whereas in the case of CSL probe there is zero intensity up to 0.4 nm. The results for DPPC‐CHOL and CHOL‐CHOL distances for relevant atoms are also presented for comparison. The difference in the interaction of CHOL with spin probes can also be visualised with the help of Voronoi diagrams of DPPC:CHOL lipid bilayers (Figure S7) calculated from simulation snapshots for two temperatures. The diagrams clearly show that in the presence of 30 % CHOL almost all 5‐PC and 16‐PC spin probes have CHOL molecules in close proximity, whereas in the case of CSL probe there are many sites lacking CSL‐CHOL contacts. The results indicate that CHOL molecules statistically prefer contacts with 5/16‐PC probes, which are structurally close to the DPPC host molecules, rather than with CSL.

This conclusion is supported further by the analysis of the hydrogen bond network formed among CHOL, phospholipids and the spin probes. The results of the calculated RDFs (Figures S8–S11) show that several hydrogen bonds are formed between CHOL and 5/16‐PC spin probes. They are similar to the ones between CHOL and DPPC and within the head group position of DPPC. For instance, strong sharp peaks in RDFs at *r* of 2.8 Å–2.9 Å, characteristic of hydrogen bonding, are observed for O3−O13, O3−O22, O3−O32 pairs of CHOL and DPPC, 5‐PC and 16‐PC molecules, respectively. As noted above the addition of CHOL increases both the effective correlation time and the order parameter of *z* magnetic axis in 5‐PC at all temperatures. This can be related to the fact that CHOL has strong hydrogen bonds with 5‐PC in the head region and therefore imposes strong steric and motional restraints on the nearby nitroxide head group leading to the decrease of both the rotational rates and orientational freedom of the nitroxide moiety. In the case of 16‐PC the interaction of CHOL with the end of acyl chain is much weaker (no hydrogen bonds formed) resulting in almost no change in the correlation times of the *z* axis of nitroxide (see Table 1). At the same time the rigidity of CHOL rings reduce the freedom of motion of acyl chains of 16‐PC leading to the increased values of the associated order parameter. CHOL is known for its dual stabilizing‐destabilising effect on lipid bilayers in the vicinity of the S_o_‐L_d_ phase transition. This makes the observation of this transition less pronounced in both experimental and predicted EPR line shapes. For instance, the effect of CHOL on preventing the ordered packing of lipids below the *T*_m_ temperature is supported by an additional MD run performed at lower temperature (*T*=283 K). Figure S12 shows that at this temperature the calculated order parameter for the *z* axis of 16‐PC probe in the presence of CHOL is significantly reduced compared to pure DPPC (from *S_0_*=0.37 to *S_0_*=0.26), which is an expected behaviour opposite to that seen in higher temperature simulations (see Figure 3 bottom panel and Table 1).

Because of the lower number of direct contacts, the impact of CHOL on the dynamics of CSL spin probe is markedly different. The main impact on CSL from the presence of CHOL is a significant reduction of the effective volume per CSL probe. As a result, the motional freedom of CSL probe is lowered and the order parameter of its main *y*‐axis is increased at each temperature while the effective rotational correlation time remains largely unaffected (Table 1).

Our results indicate that in both AA MD simulations of DPPC and DPPC:CHOL lipid bilayers several hydrogen bonds between CSL and DPPC host molecules can be formed. A small increase in the hydrogen bonding with the neighbouring DPPC molecules in the presence of CHOL is also evident form the calculated RDF of O1−C22 and O1−C32 pairs of CSL and DPPC molecules (Figure S13). It is worth noting that the flip‐flop of CSL molecules occurs in MD simulations at every temperature in the absence of CHOL. Interestingly though, CSL spin probes inserted in DPPC:CHOL lipid bilayers do not experience any flip‐flop during MD runs at all temperatures. In the simulations 5‐PC and 16‐PC spin probes are not subjected to flip‐flop at all temperatures both in the absence and in the presence of CHOL.

Overall, hydrogen bonds formed between CHOL and 5/16‐PC reduce both the motional space and rotational speeds of the nearby nitroxide in 5‐PC, while in the case of 16‐PC only the motional space of the nitroxide moiety is reduced. On the other hand, CHOL has a more indirect impact on CSL by promoting stronger packing with the surrounding DPPC molecules leading to a reduction in motional freedom for the nitroxide head group.

### 2.6 Correlation Between the Motions of Spin Probes and Associated Parts of Phospholipids and Cholesterol

It is instructive to analyse the correlations between the motions of the nitroxide group in each spin probe and the associated part of the DPPC molecules, and the impact of CHOL and temperature on these correlations.

Table 2 lists rotational correlation times and order parameters, in the absence and presence of CHOL, of *z* and *y* axes of molecular frames attached at positions C25 and C216 of the sn‐2 chains of DPPC as defined in Figure 1. Similar parameters are also provided for the CHOL molecule (atom C3). In the case of C25 and C216 atoms the direction of *y* axis is defined along one of the C−H bonds while the *z* axis is calculated as a vector product between the two adjacent C−H bonds. For the C3 atom of CHOL the *z* and *y* axes are defined along the associated C−H and C−O bonds, respectively.

**Table 2:** Effective correlation times and order parameters of the relevant molecular axes of C25 (DPPC), C216 (DPPC) and C3 (CHOL).

*T*(K)	$\tau_{eff}^{y}$ (ns)	$\tau_{eff}^{z}$ (ns)	$S_{0}^{a}$	$\tau_{eff}^{y}$ (ns)	$\tau_{eff}^{z}$ (ns)	$S_{0}^{a}$	$\tau_{eff}^{y}$ (ns)	$\tau_{eff}^{z}$ (ns)	$S_{0}^{a}$
	C25 (DPPC)			C216 (DPPC)			C3 (CHOL)		
298	2.4 (1.0)^[a]^	3.2 (2.1)	0.69 (0.78)	0.9 (1.3)	0.05 (0.02)	0.14 (0.10)	21.7	7.6	0.46
308	0.8 (0.7)	1.8 (2.1)	0.57 (0.73)	0.7 (0.3)	0.02 (0.02)	0.08 (0.10)	15.5	6.2	0.41
313	0.6 (0.5)	1.4 (1.3)	0.46 (0.72)	0.6 (0.5)	0.01 (0.01)	0.04 (0.09)	13.4	1.8	0.40
318	0.8 (0.5)	1.0 (1.0)	0.45 (0.68)	1.4 (0.5)	0.01 (0.01)	0.03 (0.08)	11	1.4	0.37
333	0.7 (0.6)	0.6 (0.6)	0.40 (0.59)	1.2 (0.6)	0.01 (0.01)	0.01 (0.05)	9.2	0.4	0.36

$S_{0}^{a}$ represents order parameters for the *z*‐ and *y*‐ molecular axes attached at positions C25/C216 of DPPC and C3 of CHOL, respectively (see Figure 1b), estimated from the fitting of associated autocorrelation functions; [a] values in parentheses are for the DPPC:CHOL mixtures.

**Figure 5:**
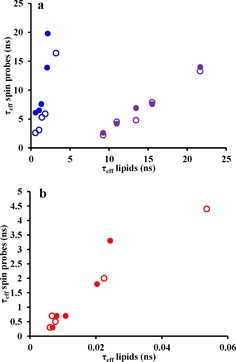
Relationship between rotational correlation times of spin probes and associated parts of lipids and cholesterol at different temperatures. The rotational correlation times increase as the temperature decreases from 333 K to 298 K. a) Correlation times of magnetic vectors *z* of 5‐PC and y of CSL versus their molecular counterparts are shown in blue and purple, respectively. b) Correlation times of magnetic vector *z* of 16‐PC vs. its molecular counterpart are shown in red. In each panel open and closed symbols represent correlation times in DPPC and DPPC:CHOL lipid bilayers, respectively.

Figure 5 compares the correlation times of the magnetic *z* vectors of 5/16‐PC spin probes and *z* vectors of the associated C−H_2_ in the sn‐2 chain of DPPC as well as of magnetic *y* vector of the CLS spin probe and the *y* vector of CHOL for the simulated temperatures. A linear correlation is observed between the correlation times of the probes and their associated counterparts.

According to Table 2 the order parameters of molecular *z* vectors at positions C25 and C216 of the carbon chain are both increased with the addition of CHOL. In the presence of CHOL the values of the order parameters for 5‐PC probe and the one at C25 of DPPC are both high and close to each other, while the values of the order parameters of 16‐PC spin probe and the one at C216 of DPPC are both small, exhibiting lower alignment with the bilayer normal.

This different behaviour is due to the CHOL‐induced ordering of the top half of the acyl chains of both DPPC and 5/16‐PC, which decreases the off‐axial rotation because the ring system induces a greater tendency for *trans* bonds; simultaneously, CHOL generally increases the flexibility of the bottom half of the acyl chains by allowing free volume for the *gauche* bonds. These observations are in good agreement with recently published experimental results.11c,^32^ CHOL itself maintains relatively high values of the order parameter (*y* axis defined as the C3−O3 bond) for every temperature (Table 2). These values are close to the ones calculated for the CSL probe (e. g. 0.46 and 0.55 for CHOL and CSL, respectively, at 298 K and 0.36 and 0.37 for CHOL and CSL, respectively, at 333 K). This is attributed to structural similarity between the two molecules.

The situation is somewhat different when correlation times of the magnetic axes of 5/16‐PC spin probes and the relevant sites of the sn‐2 carbon chain are compared. As seen from Table 2 and Figure 5, although the correlation times of *z* and *y* vectors at C25 and C216 are progressively reduced with increasing temperature they are not affected much by the addition of CHOL. This behaviour is opposite to that exhibited by the 5‐PC probe, where correlation times are significantly increased in the presence of CHOL, but similar to the behaviour observed for the 16‐PC probe. This difference in motion between 16‐PC and 5‐PC upon addition of CHOL is attributed to the formation of multiple hydrogen bonds between CHOL and 5‐PC in the proximity of the nitroxide group slowing down its re‐orientational dynamics. Interestingly, the values of correlation time for the *y* (C3−O3) axis of CHOL are significantly larger at each simulated temperature as compared to the values for the *y*‐axis of CSL (e. g. 21.7 ns for CHOL versus 13.3 ns for CSL at 298 K and 9.2 ns for CHOL versus 2.2 ns for CSL at 333 K). This difference is most likely due to the hydrogen bonds that CHOL forms with the head groups of the DPPC molecules resulting in slow tilting motions of the former.

## 3 Conclusions

In summary, this study reports the first prediction of the VT EPR spectra of lipid bilayers in the absence and presence of CHOL doped with structurally different spin probes directly and completely from state‐of‐the‐art MD simulations. Such an approach offers two important advances. Firstly, it provides a direct assignment and interpretation of EPR spectra from MD trajectories of actual structures with motions that are accounted for explicitly. Secondly, the MD‐EPR simulation methodology serves as a rigorous test bed for current MD models against an experimental technique that is highly sensitive to molecular organisation and motions. Large time/size scale all‐atom MD simulations have been performed on DPPC and DPPC:CHOL lipid bilayer systems employing the latest version of Slipids force‐field parameters.^18^ This allowed us to use multiple spin probes for enhanced sampling of their motions and concatenated MD trajectories of relatively long lengths for prediction of the EPR line shapes. Our results show very good agreement with experiments broadly confirming the accuracy of the latest force‐fields developed for lipids.

The advantage of this direct MD‐EPR simulation approach over previous ones is that it explicitly accounts for the complexity of both local and global motions of both the probes and the host phospholipids. We demonstrate direct and indirect effects of CHOL on the motions and order of structurally different spin probes and the role that hydrogen bonds play in such interactions. Analysis indicates strong linear regression between re‐orientational motions of the nitroxide group in spin probe molecules and associated parts in phospholipid and cholesterol molecules upon varying the temperature.

The reported MD‐EPR simulation approach to biological membranes allows explicit relationship between sensitive EPR line shapes and molecular motions and organisation in lipid bilayers with atomistic resolution. Importantly, it eliminates the ambiguity of interpretation of EPR spectra in the previous methods that relied on the fitting of spectra with multiple adjustable parameters. The approach presented here would be particularly valuable in the investigation of complex phenomena such as lipid domain aggregation in ternary lipid systems, including miscibility critical points on the phase diagrams and the formation of lipid rafts. This work is currently in progress.

## Conflict of interest

The authors declare no conflict of interest.

## Supporting information

As a service to our authors and readers, this journal provides supporting information supplied by the authors. Such materials are peer reviewed and may be re‐organized for online delivery, but are not copy‐edited or typeset. Technical support issues arising from supporting information (other than missing files) should be addressed to the authors.

Supplementary
