# Supplementary material for: Direct Prediction of EPR Spectra from Lipid Bilayers: Understanding Structure and Dynamics in Biological Membranes
Source: Chemphyschem. 2018 Jun 19;19(17):2183–93. doi: 10.1002/cphc.201800386 (PMC6175124; doi:10.1002/cphc.201800386)
Supplement: Supplementary file 1 — Supplementary [file CPHC-19-2183-s001.pdf]

Supporting Information

© Copyright Wiley-VCH Verlag GmbH & Co. KGaA, 69451 Weinheim, 2018

Direct Prediction of EPR Spectra from Lipid Bilayers: Understanding Structure and Dynamics in Biological Membranes

Andrea Catte, Gaye F. White, Mark R. Wilson, and Vasily S. Oganesyan*

MD simulations details (system preparation)

All-atom (AA) DPPC and DPPC:CHOL lipid bilayers were generated with the CHARMM-GUI membrane builder [1-4], a web based graphical user interface to create input files for AA and coarse grained (CG) MD simulations of lipid bilayers with different lipid compositions. Firstly, a CG MD simulation was performed in order to achieve properly equilibrated DPPC:CHOL lipid bilayer structures with the correct phase behaviour. The AA DPPC:CHOL lipid bilayer, which contained 1200 lipids with a CHOL molar concentration of 30%, was coarse grained and CG MD simulated for 40 μs at 298 K and 1 atm using the CG MARTINI force field for lipids [5] and Gromacs version 4.5.5 [6] on 32 processor cores. The standard cutoffs of the MARTINI force field were used for non-bonded interactions: the Lennard-Jones potential was shifted to zero between 0.9 and 1.2 nm, and the Coulomb potential was shifted to zero between 0 and 1.2 nm with a relative dielectric constant of 15. The time step was 20 fs, and the neighbour list was updated every 10 steps [7]. Lipids and water were coupled separately to a target temperature using the velocity rescaling thermostat [8] with a time constant of 1 ps. A target surface tension was maintained using the surface tension coupling scheme and the Berendsen barostat [9] with a time constant of 4 ps and a compressibility of 5×10^{-5} bar^{-1} in the lateral direction; the compressibility in the normal direction was set to zero to prevent box contraction. About 5% of the CG water beads were replaced with antifreeze water particles [5] to prevent the crystallization of water [7]. Then, the 40 μs structure of the DPPC:CHOL lipid bilayer was fine grained and scaled down to a system containing 600 lipids by removing a proper amount of DPPC and CHOL molecules in order to reach a CHOL molar concentration of 30%. The initial structures of DPPC lipid bilayers containing 600 lipids and different spin probes were generated from last structures of 100 ns AA MD simulations of DPPC lipid bilayers with a low concentration of CSL and 16-PC spin probes (0.33 mol %) performed at temperatures ranging from 283 to 333 K.

5-PC and 16-PC spin probe molecules were inserted in each DPPC lipid bilayer without and with CHOL by replacing an equivalent number of DPPC molecules per leaflet in non-overlapping positions in order to reach a final concentration of the spin probe of 2 mol % and a total of 600 lipids (including spin probe molecules). CSL spin probe molecules were inserted in each DPPC lipid bilayer without CHOL by replacing DPPC molecules in the same way used for 5-PC and 16-PC spin probes. The insertion of CSL spin probes in each DPPC lipid bilayer with CHOL involved the replacement of an equivalent number of CHOL molecules in order to reach a final concentration of the spin probe of 2 mol % and a total of 600 lipids (including spin probe molecules).

The area per lipid and the bilayer thickness of DPPC and DPPC:CHOL lipid bilayers were estimated using APL@Voro version 3.0 [10]. The volume per lipid, V_L, was estimated with the following relation:

$$V_L = AD/2 - N_w V_w \qquad \text{(S1)}$$

where A is the area per lipid, D is the height of the simulation box, N_w is the number of water molecules per lipid and V_w is the volume of a water molecule in the water region of the density profile. In our simulations, $N_w = 30$, and D is a variable depending on the temperature and the presence of CHOL. We use a V_w value of 0.0305 nm^3 for every simulated system, as previously reported by Petrache et al. in 1997 [11] for a DPPC lipid bilayer at 323 K; the same V_w value was also recently used by Mukhopadhyay et al. in 2004 [12].

Further details of MD-EPR simulations

A trajectory based method that employs the numerical solution of the Stochastic Liouville Equation (SLE) in the Langevin form for the spin density matrix has been used for the simulation of CW EPR line shapes. At X-band the spectrum is dominated by the anisotropic

A tensor and, under the condition of intermediate field approximation, the three hyperfine coupling lines

$$\omega^m(t) = \frac{\left(g^L_{ZZ}(\Omega(t))\beta B + m\sqrt{A^L_{XZ}(\Omega(t))^2 + A^L_{YZ}(\Omega(t))^2 + A^L_{ZZ}(\Omega(t))^2}\right)}{h} - \omega_0 \quad \text{(S2)}$$

are the functions of the calculated dynamical trajectory $\Omega(t)$ where the relevant elements of the $\mathbf{g}^L$ and $\mathbf{A}^L$ tensors in the laboratory frame are determined from the principle values for **g** and **A** tensors [13,14] in the frame of the nitroxide using the following Cartesian transformations:

$$\mathbf{g}^L(\Omega(t)) = R(\Omega(t)) \cdot \mathbf{g} \cdot R(\Omega(t))^{-1} \quad \text{(S3)}$$

$$\mathbf{A}^L(\Omega(t)) = R(\Omega(t)) \cdot \mathbf{A} \cdot R(\Omega(t))^{-1} \quad \text{(S4)}$$

Here, $R(\Omega(t))$ defines the orientation of the nitroxide fixed magnetic axes in laboratory frame. Also, ω_0, β, h and B are resonance frequency, Bohr magneton, Planck's constant and magnetic field, respectively and $m = \pm 1, 0$. Note that there is no hyperfine contribution to the central line (m = 0). In all simulations the following principle values of **g** and **A** hyperfine coupling tensors for CSL, 5-PC and 16-PC spin probes have been employed: for CLS: g_{xx}=2.0089; g_{yy}=2.0061; g_{zz}=2.0025; A_{xx}=5.8 G; A_{yy}=5.8 G; A_{zz}=35.0 G; for 5-PC: g_{xx}=2.0089; g_{yy}=2.0061; g_{zz}=2.0025; A_{xx}=5.6 G; A_{yy}=5.0 G; A_{zz}=33.0 G; for 16-PC: g_{xx}=2.0089; g_{yy}=2.0058; g_{zz}=2.0021; A_{xx}=4.9 G; A_{yy}=4.9 G; A_{zz}=31.5 G. They are in agreement with the previously reported values by other researchers [15]. Homogeneous line broadening was taken into account with the relaxation parameter T_2 = 0.15 μs and T_2 = 0.26 μs, for 298 K, 308 K and 313 K, 318 K, 333 K, respectively.

References:

(1) Jo, S., Kim T, Iyer VG, Im W *J Comput Chem* **2008**, *29*, 1859.
(2) Brooks, B., Brooks III CL, MacKerell Jr AD, Nilsson L, Petrella RJ, Roux B, Won Y, Archontis G, Bartels C, Boresch S, Caflisch A, Caves L, Cui Q, Dinner AR, Feig M, Fischer S, Gao J, Hodoscek M , Im W, Kuczera K, Lazaridis T, Ma J, Ovchinnikov V, Paci E, Pastor RW, Post CB, Pu JZ, Schaefer M, Tidor B, Venable RM, Woodcock HL, Wu X, Yang W, York DM, Karplus M *J Comput Chem* **2009**, *30*, 1545.
(3) Wu, E., Cheng X, Jo S, Rui H, Song KC, Dávila-Contreras EM, Qi Y, Lee J, Monje-Galvan V, Venable RM, Klauda JB, Im W *J Comput Chem* **2014**, *35*, 1997.
(4) Jo, S., Lim JB, Klauda JB, Im W *Biophys J* **2009**, *97*, 50.
(5) Marrink, S., Risselada HJ, Yefimov S, Tieleman DP, de Vries AH *J Phys Chem B* **2007**, *111*, 7812.
(6) Pronk, S., Páll S, Schulz R, Larsson P, Bjelkmar P, Apostolov R, Shirts MR, Smith JC, Kasson PM, van der Spoel D, Hess B, Lindahl E *Bioinformatics* **2013**, *29*, 845.
(7) Baoukina, S., Mendez-Villuendas E, Bennett WFD, Tieleman DP *Faraday Discuss* **2013**, *161*, 63.
(8) Bussi, G., Donadio D, Parrinello M *J Chem Phys* **2007**, *126*, 014101.
(9) Berendsen, H., Postma JPM, van Gunsteren WF, DiNola A, Haak JR *J Chem Phys* **1984**, *81*, 3684.
(10) Lukat, G., Krüger J, Sommer B *J Chem Inf Model* **2013**, *53*, 2908.
(11) Petrache, H., Feller SE, Nagle JF *Biophys J* **1997**, *70*, 2237.
(12) Mukhopadhyay, P., Monticelli L, Tieleman DP *Biophys J* **2004**, *86*, 1601.
(13) Steinhoff, H.-J., Hubbell W L *Biophys J* **1996**, *71*, 2201.
(14) Oganesyan, V., Kuprusevicius E, Gopee H, Cammidge AN, Wilson MR *Phys Rev Lett* **2009**, *102*, 013005.
(15) Smith, A. K.; Freed, J. H. *Chem Phys Lipids* **2012**, *165*, 348.

The phase behaviour of DPPC in the absence and in the presence of cholesterol (CHOL) was studied. At a CHOL molar concentration of 30% the gel (S_o) phase of DPPC is destabilised at temperatures below the gel-liquid crystalline phase transition temperature and the formation of a liquid ordered (L_o) phase is observed as reported in previous experiments. The 100 ns AA MD simulation snapshots at different temperatures highlight an increase of the general disorder in DPPC lipid bilayers with increasing temperature (Fig. S1). The bilayer thickness decreases as the temperature is increased for DPPC lipid bilayers from 0.43 nm at 283 K to 0.37 nm at 333 K, indicating also that the lipid bilayer is becoming more disordered. DPPC:CHOL lipid bilayers exhibit a less pronounced decrease of the bilayer thickness from 0.45 nm at 283 K to 0.42 nm at 333 K (Figs. 2 and S2). The increased order of DPPC:CHOL lipid bilayers is also confirmed by the deuterium order parameters profiles of DPPC sn-1 and sn-2 acyl chains (Fig. S3), which are in agreement with simulated and experimental values reported previously [16-19].

References:

(16) Chiu S, Jakobsson E, Mashl RJ, Scott HL, **2002,** *Biophys J* 83:1842-1853.

(17) Jämbeck J, Lyubartsev AP, **2013,** *J Chem Theory Comput* 9:774-784.

(18) Jämbeck J, Lyubartsev AP, **2012,** *J Phys Chem B* 116(10):3164-3179.

(19) Pitman M, Suits F, MacKerell AD Jr, Feller SE, **2004**, *Biochemistry* 43:15318-15328.

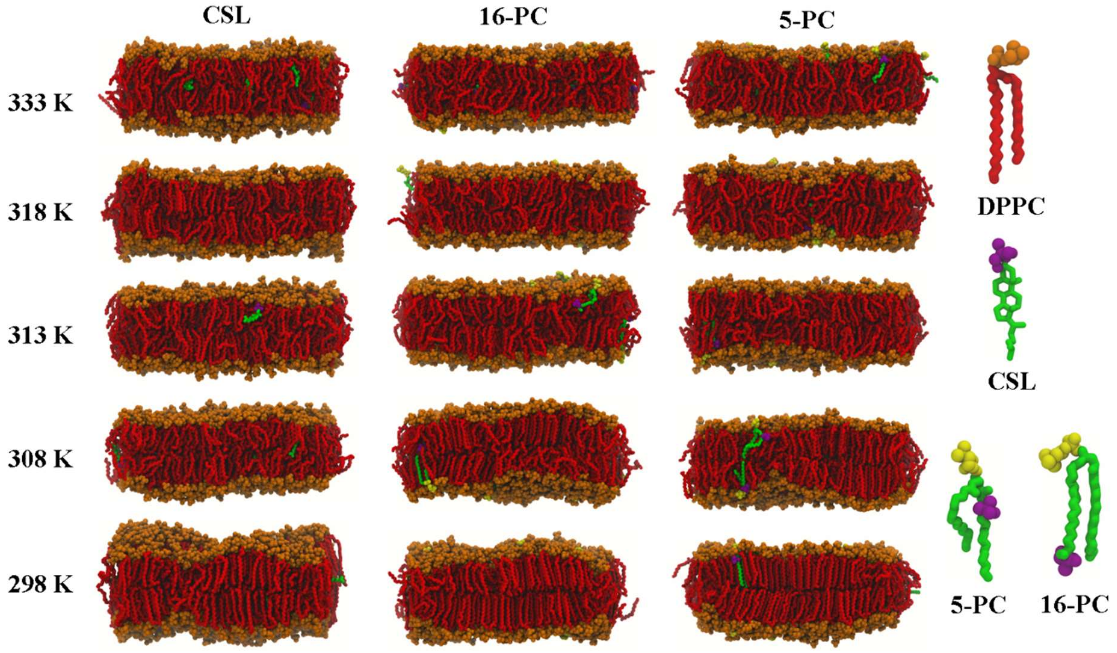

Figure S1 Side views of equilibrated structures of DPPC lipid bilayers doped with 5-PC, 16-PC and CSL spin probes simulated at different temperatures. DPPC polar head groups and hydrophobic tail groups are shown in orange space filling and red representation, respectively. 5-PC and 16-PC polar head groups are shown in yellow. Hydrophobic acyl chains and glycerol backbones of n-PC spin probes, and sterol rings and alkyl chains of CSL are shown in green. Nitroxide moieties of each spin probe are shown in purple. Hydrogens, waters and ions are not shown.

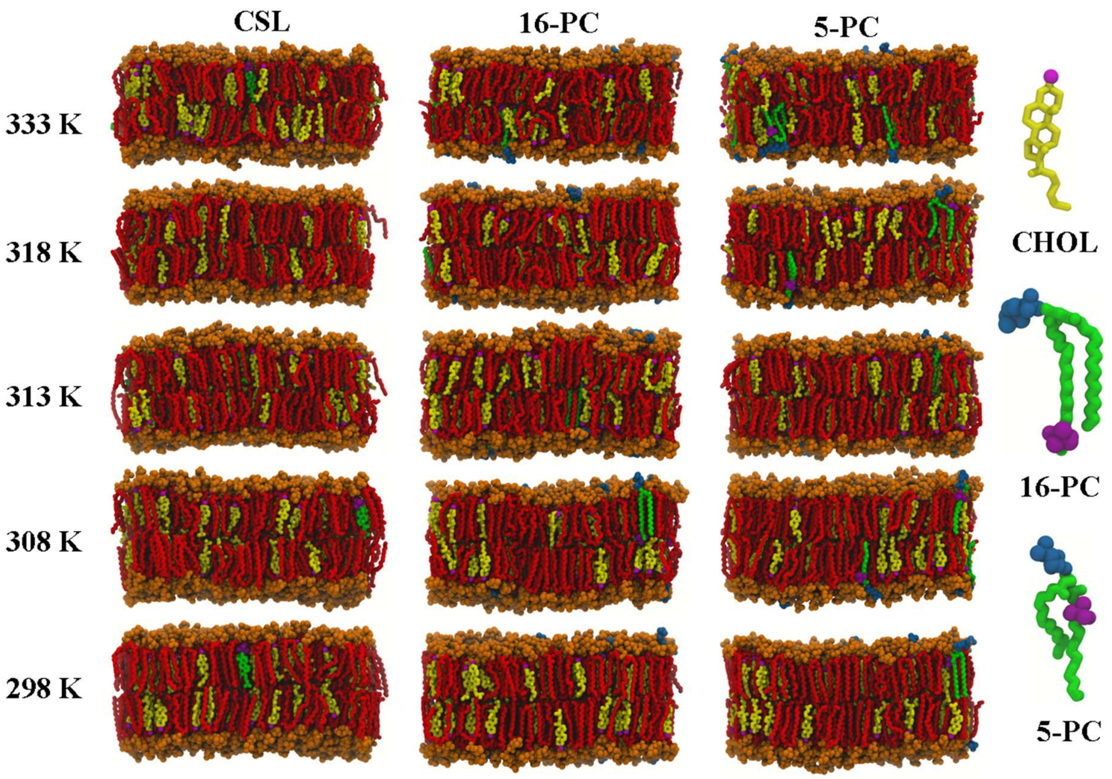

Figure S2 Side views of equilibrated structures of DPPC:CHOL lipid bilayers doped with 5-PC, 16-PC and CSL spin probes simulated at different temperatures. DPPC polar headgroups and hydrophobic tailgroups are shown in orange space filling and red representation, respectively. Oxygens of CHOL are shown in magenta representation. Sterol rings and alkyl chains of CHOL are shown in yellow. 5-PC and 16-PC polar headgroups are shown in skyblue. Hydrophobic acyl chains and glycerol backbones of n-PC spin probes, and sterol rings and alkyl chains of CSL are shown in green. Nitroxide moieties of each spin probe are shown in purple. Hydrogens, waters and ions are not shown.

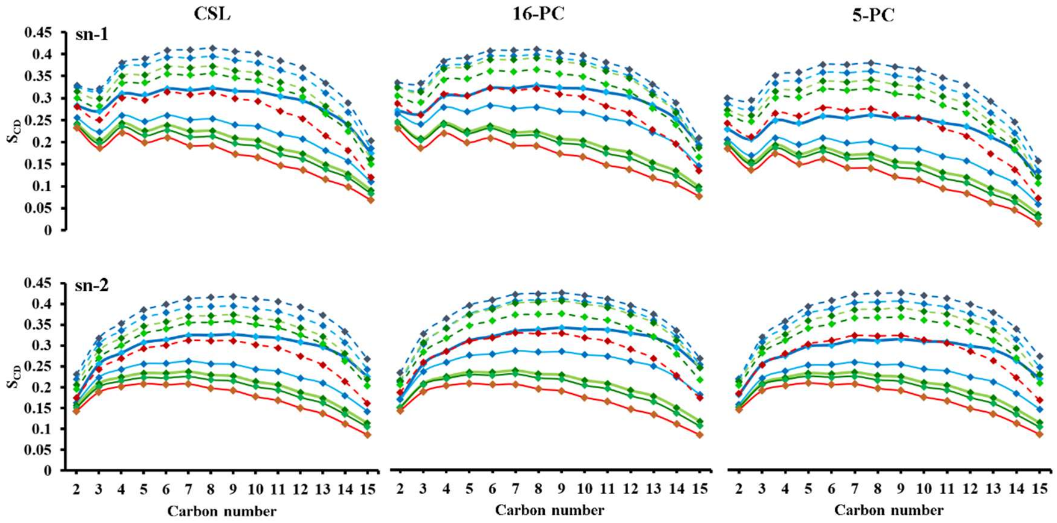

Figure S3 Order parameter profiles of DPPC sn-1 and sn-2 acyl chains in DPPC (straight lines) and DPPC:CHOL (dashed lines) lipid bilayers doped with different spin probes at different temperatures. The increase of the temperature is shown with differently coloured lines. A decrease of the order parameters is observed from 298 K (blue line) to 333 K (red line), while at a given temperature an increase of S_{CD} is observed in the presence of CHOL.

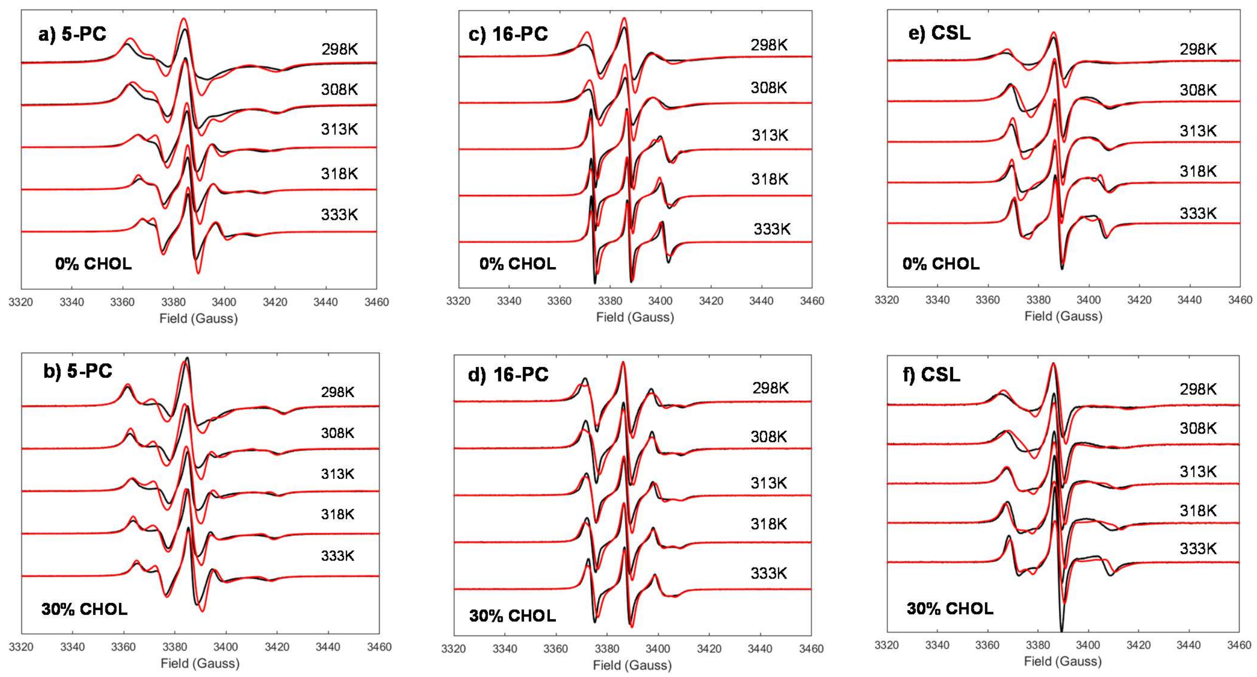

Figure S4 Comparison between predicted from MD (red lines) and experimental (black lines) EPR spectra of DPPC and DPPC:CHOL lipid bilayers doped with 5-PC (a) and b)), 16-PC (c) and d)) and CSL (e) and f)) spin probes at different temperatures. All spectra are normalised by the value calculated by double integration of the EPR line shape.

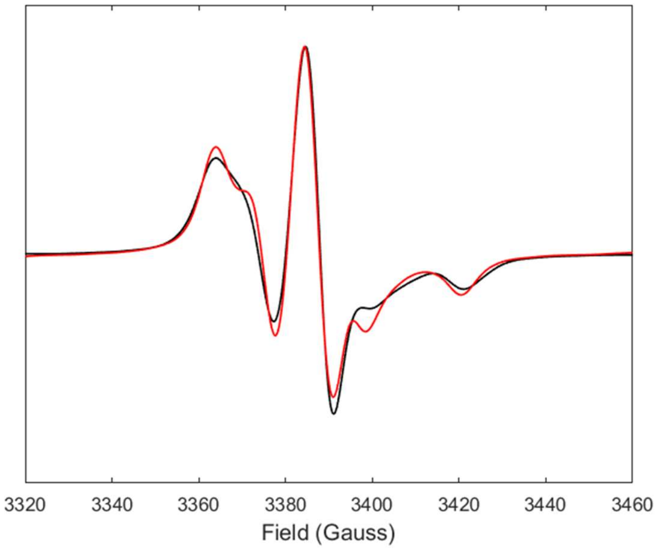

Figure S5 Comparison between EPR spectra predicted from two different 1.2 μs MD trajectories corresponding to 5-PC spin probe in DPPC at 308K.

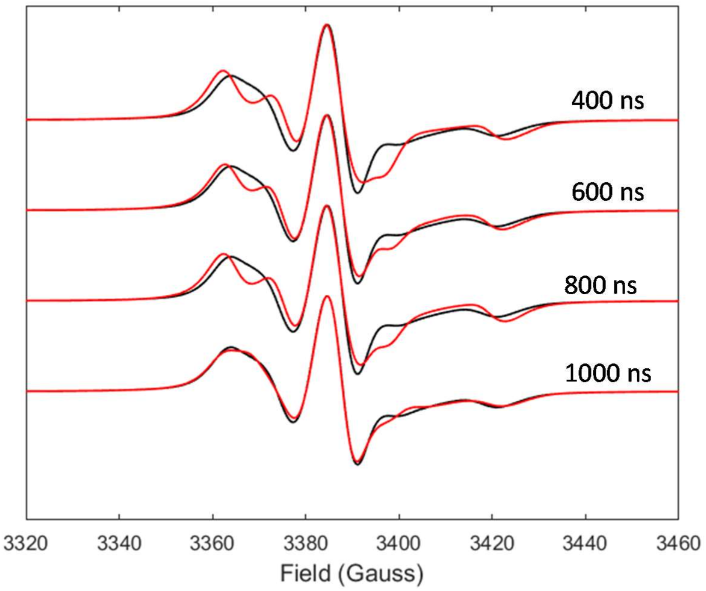

Figure S6 Convergence of the simulated EPR line shape corresponding to 5-PC spin probe in DPPC at 308K based on the use of different lengths of the MD trajectory. Red lines from top to bottom correspond to 400 ns, 600 ns, 800 ns and 1000 ns trajectories, respectively. Black lines represent the full 1200 ns MD trajectory.

--

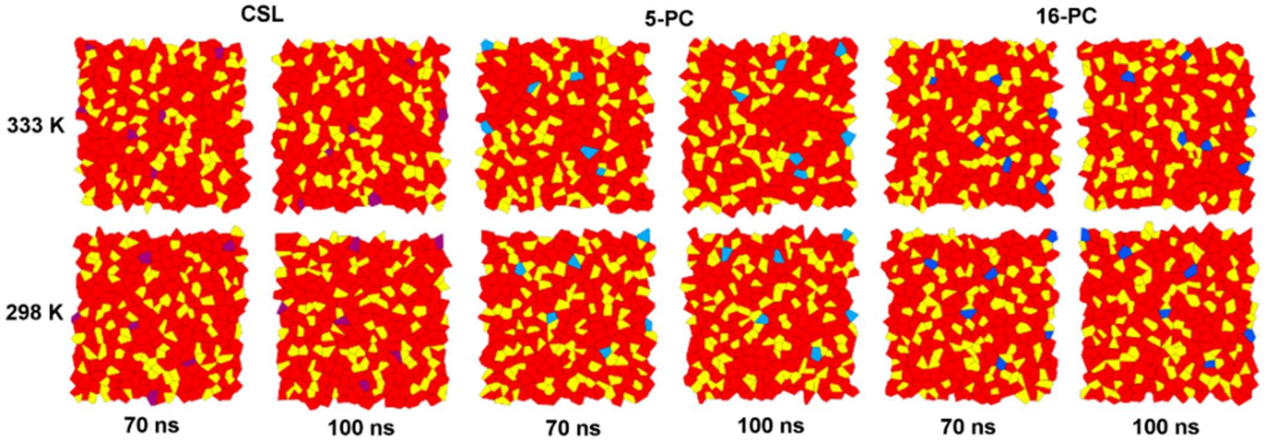

Figure S7 Voronoi diagrams of selected snapshots of DPPC:CHOL lipid bilayers showing relative positions of CSL, 5-PC, 16-PC, CHOL and DPPC molecules, shown in purple, skyblue, blue, yellow and orange, respectively, at 298 K and 333 K.

For convenience in identifying the positions of different atoms reported in the Figures S5-S8, molecular structures of DPPC, CHOL and three spin probes are reproduced here from the main text (Figure 1b). For DPPC and CHOL associated molecular vectors are given in blue. For the spin probes the magnetic axes of the nitroxide moieties are indicated in black.

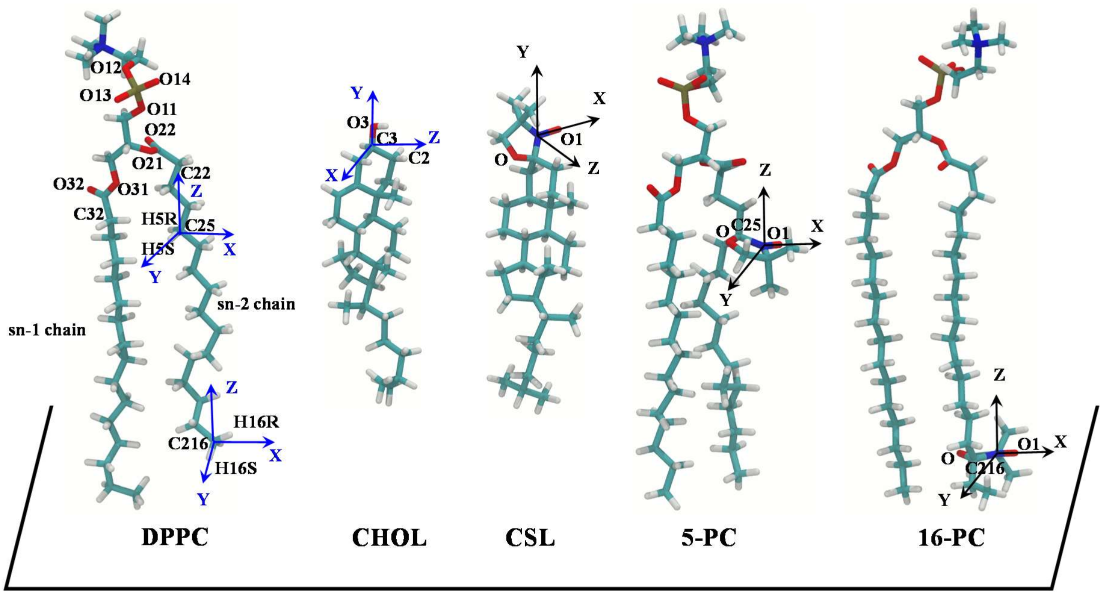

The interaction of CHOL molecules with DPPC lipids can be evaluated from the calculated radial distribution functions (RDFs) of different atom pairs, such as CHOL oxygen-DPPC carbonyl oxygens and CHOL oxygen-DPPC phosphate oxygens. They are similar to the results previously reported in the literature[16] and indicate a strong hydrogen bonding between CHOL hydroxyl groups and DPPC carbonyl oxygens without showing any specificity for the acyl chain (Fig. S5 shows the interaction with sn-1 and sn-2 chains of DPPC). Moreover, direct CHOL-CHOL interactions are confirmed by the RDFs of CHOL oxygens pairs showing peaks at 0.5, 1 and 1.5 nm at every simulated temperature (Fig. S6) and are in agreement with published results [16,19].

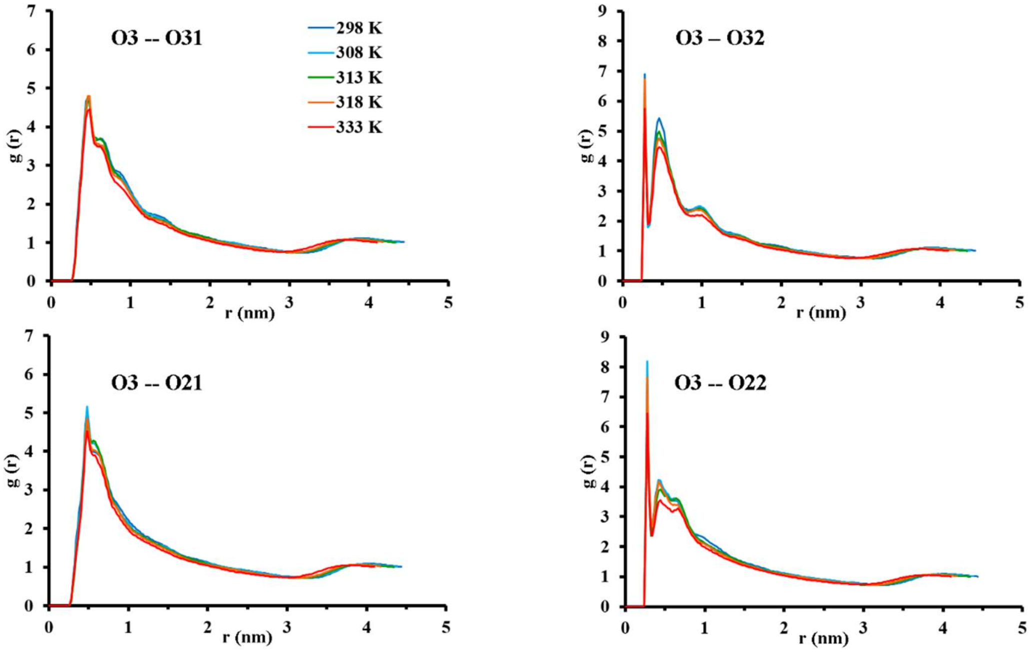

Figure S8 Radial distribution functions (RDFs) of O3-O31 O3-O32, O3-O21, and O3-O22 atom pairs of CHOL and DPPC molecules at different temperatures show the formation of strong hydrogen bonds between hydroxyl groups of CHOL molecules and ester oxygens of sn-1 (O32) and sn-2 (O22) of DPPC molecules. RDFs were calculated over the last 40 ns of each simulation of DPPC:CHOL lipid bilayers.

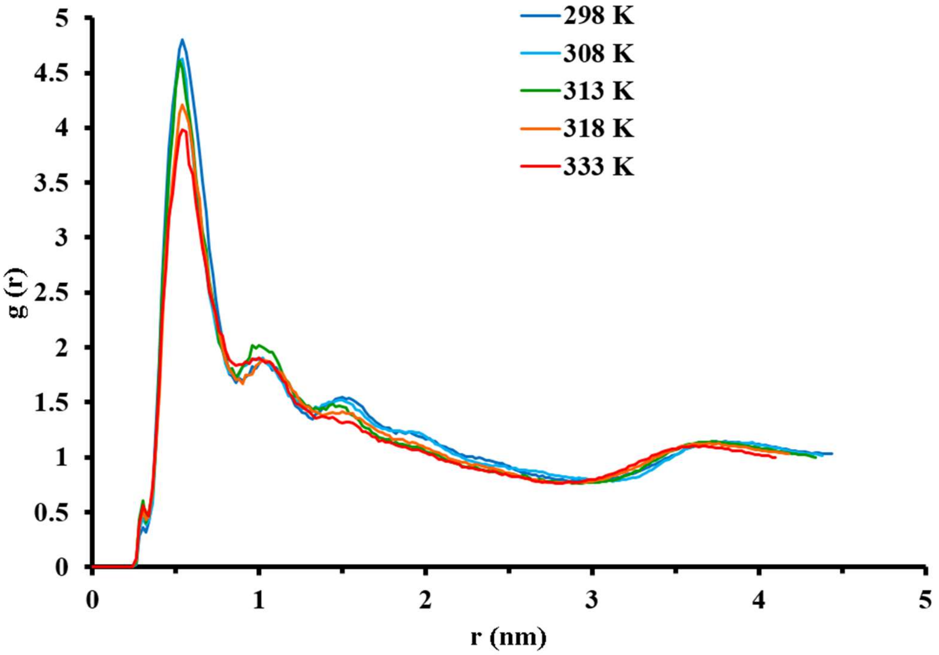

Figure S9 Radial distribution functions (RDFs) of O3-O3 atom pairs of CHOL molecules at different temperatures show the characteristic packing of CHOL in a DPPC:CHOL lipid bilayer. RDFs were calculated over the last 40 ns of each simulation.

Table S1. Correlation times, weights and order parameters of magnetic *y*-axis of CSL and magnetic *z*-axes of 5-PC and 16-PC spin probes, respectively, (bi-exponential fitting).

T (K)	w_1	τ_1 (ns)	w_2	τ_2 (ns)	τ_{eff} (ns)	S_0
			5-PC			
298	0.21 (0.29)[a]	0.5 (2.0)	0.79 (0.71)	20.4 (27.2)	16.4 (19.8)	0.65 (0.73)
308	0.38 (0.51)	1.0 (2.1)	0.62 (0.49)	8.8 (26.4)	5.9 (13.9)	0.48 (0.71)
313	0.29 (0.47)	0.7 (1.9)	0.71 (0.53)	7.2 (12.6)	5.3 (7.6)	0.49 (0.65)
318	0.45 (0.55)	1.0 (1.6)	0.55 (0.45)	4.9 (12.5)	3.1 (6.5)	0.51 (0.66)
333	0.49 (0.70)	0.5 (1.8)	0.51 (0.30)	4.6 (16.0)	2.6 (6.1)	0.41 (0.52)
			16-PC			
298	0.59 (0.66)	0.9 (0.9)	0.41 (0.34)	9.5 (8.0)	4.4 (3.3)	0.20 (0.30)
308	0.80 (0.78)	0.8 (0.7)	0.20 (0.22)	7.1 (5.6)	2.0 (1.8)	0.17 (0.24)
313	0.80 (0.53)	0.3 (0.2)	0.20 (0.47)	2.7 (1.2)	0.7 (0.7)	0.14 (0.20)
318	0.81 (0.57)	0.2 (0.2)	0.19 (0.43)	1.7 (1.2)	0.5 (0.7)	0.10 (0.17)
333	0.83 (0.86)	0.1 (0.2)	0.17 (0.14)	1.1 (1.2)	0.3 (0.3)	0.09 (0.17)
			CSL			
298	0.10 (0.32)	0.4 (2.1)	0.90 (0.68)	14.7 (19.7)	13.3 (14.0)	0.55 (0.76)
308	0.17 (0.25)	1.0 (1.0)	0.83 (0.75)	9.4 (9.8)	7.9 (7.6)	0.39 (0.73)
313	0.59 (0.38)	2.2 (1.3)	0.41 (0.62)	8.5 (10.3)	4.8 (6.9)	0.39 (0.66)
318	0.36 (0.68)	0.8 (2.0)	0.64 (0.32)	6.6 (8.7)	4.5 (4.2)	0.36 (0.65)
333	0.62 (0.77)	0.9 (1.2)	0.38 (0.23)	4.3 (7.4)	2.2 (2.6)	0.34 (0.61)

[a] values in parentheses are estimated from autocorrelation functions of spin probes in DPPC:CHOL mixtures.

Table S2. Correlation times, weights and order parameters of magnetic *z*-axis of CSL and magnetic *y*-axes of 5-PC and 16-PC spin probes, respectively, (bi-exponential fitting).

T (K)	w_1	τ_1 (ns)	w_2	τ_2 (ns)	τ_{eff} (ns)
			5-PC		
298	0.19 (0.12)[a]	0.6 (0.8)	0.81 (0.88)	15.2 (14.1)	12.5 (12.5)
308	0.42 (0.23)	1.3 (1.5)	0.58 (0.77)	12.2 (13.2)	7.7 (10.5)
313	0.29 (0.30)	0.7 (1.4)	0.71 (0.70)	6.3 (14.8)	4.7 (10.8)
318	0.46 (0.31)	1.1 (1.3)	0.54 (0.69)	5.1 (13.4)	3.2 (9.6)
333	0.55 (0.38)	0.8 (0.6)	0.45 (0.62)	4.7 (3.5)	2.6 (2.4)
			16-PC		
298	0.51 (0.61)	0.7 (0.9)	0.49 (0.39)	7.2 (9.2)	3.9 (4.2)
308	0.60 (0.52)	0.4 (0.3)	0.40 (0.48)	2.2 (1.8)	1.1 (1.0)
313	0.79 (0.54)	0.2 (0.2)	0.21 (0.46)	1.5 (1.4)	0.5 (0.8)
318	0.76 (0.70)	0.2 (0.3)	0.24 (0.30)	1.1 (2.1)	0.4 (0.8)
333	0.82 (0.67)	0.1 (0.05)	0.18 (0.33)	0.8 (0.4)	0.2 (0.2)
			CSL		
298	0.30 (0.42)	0.8 (0.6)	0.70 (0.58)	16.3 (9.4)	11.6 (5.8)
308	0.48 (0.39)	0.8 (0.5)	0.52 (0.61)	7.8 (5.4)	4.4 (3.5)
313	0.61 (0.30)	0.6 (0.3)	0.39 (0.70)	5.0 (2.9)	2.3 (2.1)
318	0.66 (0.41)	0.7 (0.5)	0.34 (0.59)	5.0 (2.8)	2.2 (1.9)
333	0.62 (0.44)	0.2 (0.2)	0.38 (0.56)	2.2 (1.1)	1.0 (0.7)

[a] values in parentheses are estimated from autocorrelation functions of spin probes in DPPC:CHOL mixtures.

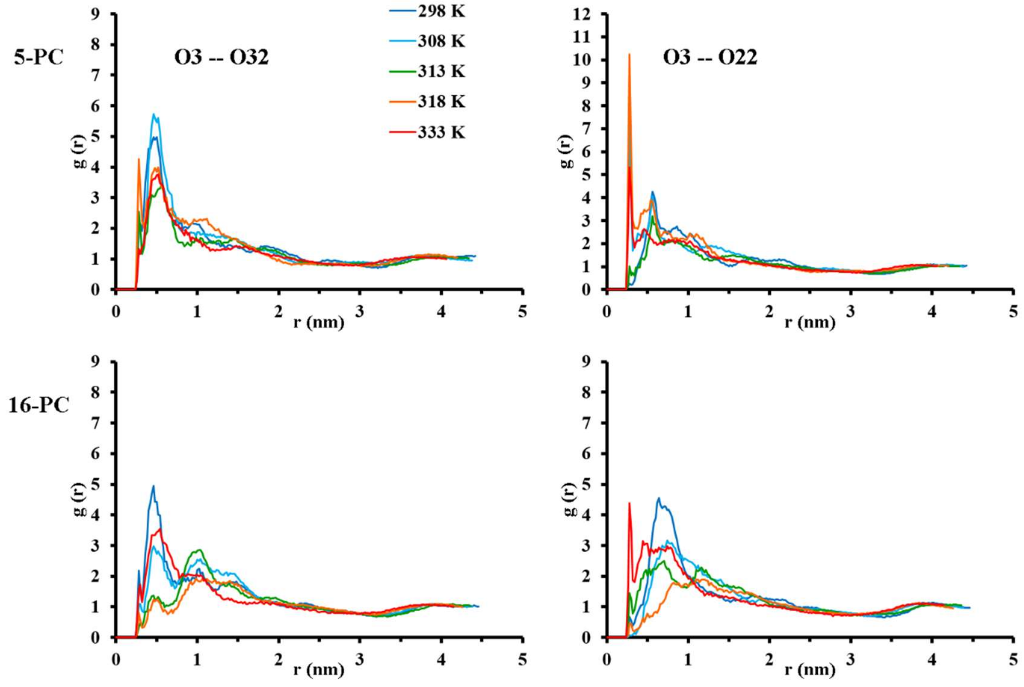

Figure S10 Radial distribution functions (RDFs) of O3-O32 and O3-O22 atom pairs of CHOL and n-PC spin probes in DPPC:CHOL lipid bilayers doped with 5-PC and 16-PC spin probes at different temperatures show the formation of hydrogen bonds similar to those observed between CHOL and DPPC molecules. RDFs were calculated over the last 40 ns of each simulation.

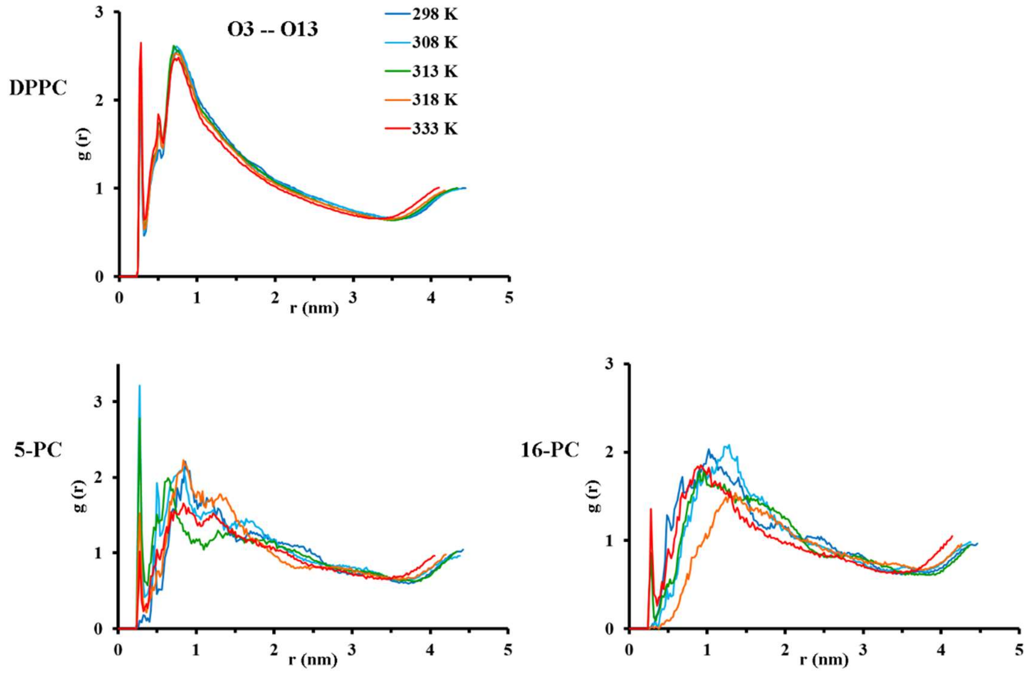

Figure S11 Radial distribution functions (RDFs) of O3-O13 atom pairs of CHOL-DPPC and CHOL-n-PC spin probes in DPPC:CHOL lipid bilayers doped with 5-PC and 16-PC spin probes at different temperatures. n-PC spin probes show the formation of hydrogen bonds of hydroxyl groups of CHOL with their phosphate oxygens (O13) similar to those observed between CHOL and DPPC molecules. RDFs were calculated over the last 40 ns of each simulation.

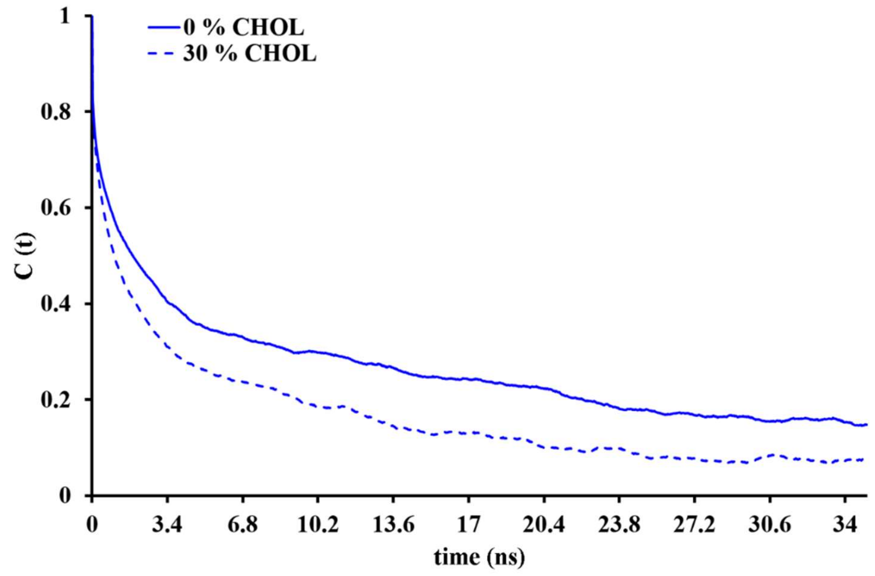

Figure S12 Autocorrelation functions of the magnetic *z*-axis of 16-PC spin probe in DPPC (solid line) and DPPC:CHOL (dashed line) lipid bilayers at 283 K.

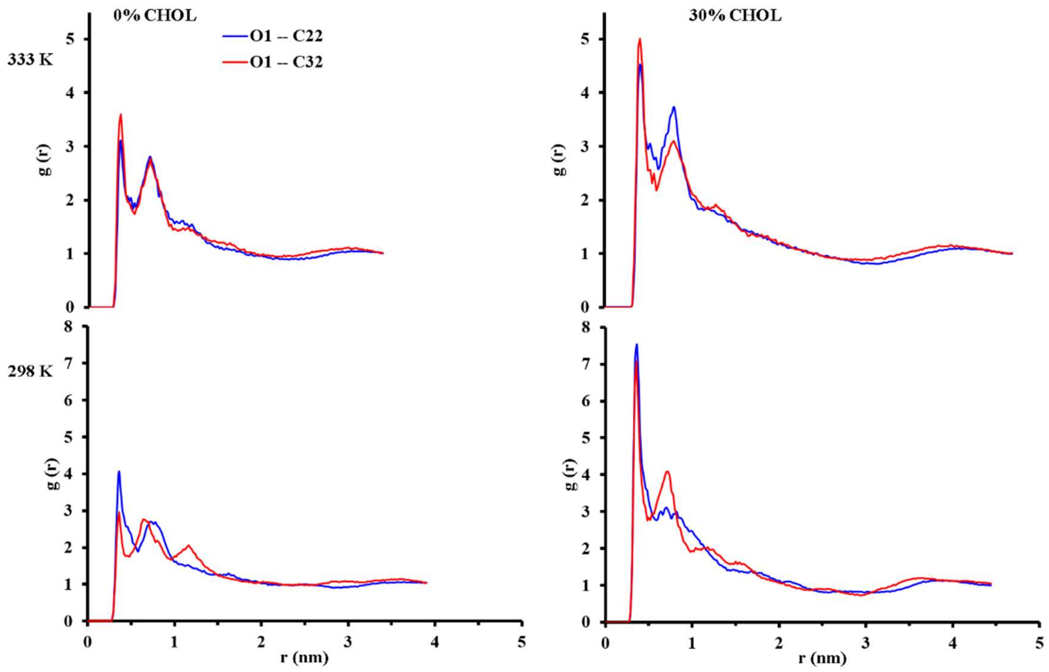

Figure S13 Radial distribution functions (RDFs) of O1-C32 and O1-C22 atom pairs of CSL and DPPC in DPPC and DPPC:CHOL lipid bilayers doped with CSL spin probes at two different temperatures. The increase of the intensity of peaks at 0.35 nm in DPPC:CHOL lipid bilayers (30% CHOL) indicates that the formation of hydrogen bonds is increased by the presence of CHOL molecules. RDFs were calculated over the last 40 ns of each simulation.
